# Fraud in highly appreciated fish detected from DNA in Europe may undermine the Development Goal of sustainable fishing in Africa

**DOI:** 10.1038/s41598-021-91020-w

**Published:** 2021-06-01

**Authors:** Carmen Blanco-Fernandez, Alba Ardura, Paula Masiá, Noemi Rodriguez, Laura Voces, Marcelino Fernandez-Raigoso, Agustín Roca, Gonzalo Machado-Schiaffino, Eduardo Dopico, Eva Garcia-Vazquez

**Affiliations:** 1 Department of Functional Biology, University of Oviedo, Oviedo, Spain; 2 Department of Education Sciences, University of Oviedo, Oviedo, Spain

**Keywords:** Genetics, Environmental sciences, Environmental social sciences

## Abstract

Despite high effort for food traceability to ensure safe and sustainable consumption, mislabeling persists on seafood markets. Determining what drives deliberate fraud is necessary to improve food authenticity and sustainability. In this study, the relationship between consumer’s appreciation and fraudulent mislabeling was assessed through a combination of a survey on consumer’s preferences (N = 1608) and molecular tools applied to fish samples commercialized by European companies. We analyzed 401 samples of fish highly consumed in Europe and worldwide (i.e. tuna, hake, anchovy, and blue whiting) through PCR-amplification and sequencing of a suite of DNA markers. Results revealed low mislabeling rate (1.9%), with a higher mislabeling risk in non-recognizable products and significant mediation of fish price between consumer´s appreciation and mislabeling risk of a species. Furthermore, the use of endangered species (e.g. *Thunnus thynnus*), tuna juveniles for anchovy, and still not regulated *Merluccius polli* hake as substitutes, points towards illegal, unreported and/or unregulated fishing from African waters. These findings reveal a worrying intentional fraud that hampers the goal of sustainable seafood production and consumption, and suggest to prioritize control efforts on highly appreciated species.

## Introduction

For the accomplishment of Goal 12 “Responsible Production and Consumption” (UN Sustainable Development Goals, SDG, https://sdgs.un.org/goals accessed in January 2021), one of the priorities in food sciences today is the traceability of the food products to be able to authenticate the species and the geographic origin. It is especially important to obtain food directly from natural populations, by hunting, fishing or harvesting. Only knowing their real exploitation level can the natural populations be sustainably managed. When a species is sold for another, the substitute is exploited without reporting. Moreover, if the substitute species is cheaper, the consumer will pay an unfair higher price for the product they believe is consumed.

Despite the obvious interest of honest labeling to warrant both the respect of consumers’ rights and resource sustainability, and increased control efforts, fish mislabeling is still happening in all continents involving species from all the seas (e.g.^1–5^. In some marine species as the Atlantic cod *Gadus morhua* mislabeling is decreasing^6^ while in others like hake it has oscillated over the last decades and seems to be increasing in the last years^7^. DNA analysis is widely used for becoming increasingly affordable, even when multiple markers are needed^1,8,9^. Not always mislabeling is synonymous of fraud. Sometimes it is accidental, for example when different species are caught together in mixed fisheries and cannot be distinguished easily from morphology, as megrims of the genus *Lepidorhombus* or several hakes of the genus *Merluccius*^10^. In other cases, however, mislabeling is deliberate so it can be considered fraudulent. In contrast with accidental mislabeling, deliberate mislabeling is more frequent in unrecognizable (versus recognizable) products^11,12^. In recognizable products the head is present and the whole body contains morphological characteristics that serve to identify the species, while the species cannot be ascertained visually in unrecognizable products. Often substitute species are cheaper than the original one^13,14^; fraudulent seafood mislabeling of economic cause happens against the interest of the consumer. Another type of fraudulent mislabeling occurs as a consequence of illegal, unreported and/or unregulated fishing (IUU); in those cases the defrauder benefits from selling inadvertently species that are often poorly managed and wild caught^15^. Examples are the covered exploitation of protected species, (e.g., high levels of mislabeling concealing endangered species have reported in sharks^16,17^, or selling individuals caught over quota as suggested in Miller et al.^18^. This is especially pernicious not only for species conservation but also for the society, because selling IUU catch undermines consumer’s awareness and conscious efforts of sustainable consumption^19^.

In the control of deliberate mislabeling, a priority is to determine the point of the commercial chain and the type of product where it happens more frequently^20^; that is to know mislabeling niches for control to be reinforced therein^21^. Is there a role of consumer’s appreciation in fish mislabeling? Apparently, it is especially frequent in highly appreciated products, like sushi^22^, processed cod products^23^, scarce and expensive Bluefin tuna *Thunnus thynnus* that is commonly replaced by other tuna species^24,25^. In other words, defrauders sell what the consumer wants whether the product is real or not. However, fraudulent mislabeling having an important economic component, defrauders will substitute preferentially, if not only, expensive species.

In this study we will focus on the relationship between consumer’s appreciation and fraudulent mislabeling in Spain. This country was chosen for having been identified as one of the countries with highest seafood consumption (42.4 kg/person/year) and also seafood mislabeling in the world^14^. We considered two species pairs of blue and white fish, one cheap and one expensive from each type. We analyzed consumer’s appreciation from self-reported consumption habits, and, using a suite of DNA markers to authenticate seafood samples, we determined mislabeling in recognizable and unrecognizable fish products.

Expectations were: First, in deliberate mislabeling a higher proportion in not recognizable versus recognizable products and expensive versus cheap species (Hypothesis *a*). Second, and central to this study, price will mediate between consumer’s appreciation and % of substitution, more expensive species being more mislabeled regardless consumer’s appreciation (Hypothesis *b*).

## Results

### Fish mislabeling

In total we analyzed genetically 401 fish samples commercialized by European companies, obtained from 11 stores. DNA extraction and PCR amplification were successful in 365 samples (91% of success): 83 labeled as anchovy (72 with the scientific name *Engraulis encrasicolus*, 11 *Engraulis* sp.), 96 as hake (40 *Merluccius merluccius*, 29 M*. hubbsi*, 27 M*. capensis/M. paradoxus*), 96 as tuna (57 *Thunnus alalunga*, 36 T*. albacares*, 3 T*. obesus*) and 90 as blue whiting (*Micromessistius poutassou*). The sequences of the genetic barcodes were aligned within species, and the haplotypes (sequence variants) submitted to GenBank, where they are available with the accession numbers MW557502-MW557516 for control region barcodes and MW549508-MW549521 for COI barcodes.

Mislabeling was found in seven samples, 1.9% of the total samples analyzed (Table 1): two of anchovy (one product per sample), two of hake (four products, one in one sample and three in other) and three of tuna (five products in total: one, two and two per sample). The total proportion of mislabeling was 0% in blue whiting, 2.4% in anchovy, 4.17% in hake and 5.2% in tuna. From the label, five of the 11 mislabeled products were caught in front of African coasts (Center-NE Atlantic, Central E Atlantic, SE Atlantic), four from European waters (NE Atlantic, Aegean Sea) and two had no geographic information in the label (Table 2).

**Table 1:** Mislabeling found in this study.

Species	Presentation	Store	Selling point	Presentation	N	Mislabeling
Anchovy	Recognizable	A	Supermarket	Unpacked	28	0.036
Anchovy	Recognizable	B	Small shop	Unpacked	9	0
Anchovy	Recognizable	C	Small shop	Unpacked	18	0
Anchovy	Recognizable	J	Small shop	Unpacked	5	0
Anchovy	Unrecognizable	D	Supermarket	Packed	22	0.045
Anchovy	Recognizable	E	Supermarket	Unpacked	1	0
Hake	Recognizable	A	Supermarket	Unpacked	14	0
Hake	Recognizable	K	Small shop	Unpacked	4	0
Hake	Recognizable	C	Small shop	Unpacked	15	0
Hake	Unrecognizable	D	Supermarket	Packed	5	0
Hake	Unrecognizable	E	Supermarket	Packed	21	0
Hake	Unrecognizable	G	Supermarket	Packed	15	0.067
Hake	Unrecognizable	E	Supermarket	Packed	17	0.176
Hake	Unrecognizable	H	Supermarket	Packed	5	0
Tuna	Unrecognizable	A	Supermarket	Unpacked	11	0.091
Tuna	Unrecognizable	C	Small shop	Unpacked	17	0.118
Tuna	Unrecognizable	L	Small shop	Unpacked	2	0
Tuna	Unrecognizable	M	Small shop	Unpacked	8	0
Tuna	Unrecognizable	E	Supermarket	Packed	35	0
Tuna	Unrecognizable	F	Fishmonger	Packed	16	0.125
Tuna	Unrecognizable	N	Small shop	Unpacked	7	0
Whiting	Recognizable	A	Supermarket	Unpacked	25	0
Whiting	Recognizable	O	Small shop	Unpacked	4	0
Whiting	Recognizable	C	Small shop	Unpacked	9	0
Whiting	Unrecognizable	I	Fishmonger	Packed	52	0

Presented by species, store, type of selling point and commercial presentation (as recognizable versus not recognizable).

**Table 2:** Mislabeled and substitute species.

Sample	Product	Information on label		Best match in GenBank–BLAST results			% Identity	Seller	Cause	Mislabeling point
		Species	Origin	Species	Accession number	Score				
96-BoT	R	*Engraulis encrasicolus*	NE Atlantic	*Thunnus albacares*	KP298900.1	558	97.26	Superstore	IUU	Ship/FM
BoCoFAI-33	U	*Engraulis encrasicolus*	Aegean Sea	*Thunnus alalunga*	KP412748.1	866	99.26	Superstore	IUU	Ship/FM
273-Bn1	U	*Thunnus alalunga*	NE Atlantic	*Thunnus thynnus*	EU562888.1	673	98.18	Local store	IUU	Ship/FM
275-Bn3	U	*Thunnus alalunga*	NE Atlantic	*Thunnus thynnus*	EU562888.1	721	97.85	Local store	IUU	Ship/FM
276-BnA	U	*Thunnus alalunga*	Center-NE Atlantic	*Thunnus thynnus*	EU562888.1	693	99.22	Superstore	IUU	Ship/FM
BnCBsN-22.2	U	*Thunnus alalunga*	NA	*Thunnus albacares*	AF301199.1	809	99.11	Superstore	IUU	Ship/FM
BnCBsN-22.3	U	*Thunnus alalunga*	NA	*Thunnus obesus*	KJ535743.1	472*	88.1	Superstore	IUU	Ship/FM
MCoFMer-2	U	*Merluccius capensis/paradoxus*	SE Atlantic	*Gadus morhua*	MT456169.1	588	100	Superstore	IUU/Economy	FM/SELL
Mc-02	U	*Merluccius capensis/paradoxus*	Central E Atlantic	*Merluccius polli*	EF362877.1	708	100	Superstore	IUU/Economy	FM/SELL
Mc-03	U	*Merluccius capensis/paradoxus*	Central E Atlantic	*Merluccius polli*	EF362877.1	763	99.76	Superstore	IUU/Economy	FM/SELL
Mc-04	U	*Merluccius capensis/paradoxus*	SE Atlantic	*Merluccius polli*	EF362877.1	754	99.52	Superstore	IUU/Economy	Ship/FM/SELL

Sample code, product type (R: recognizable, U: unrecognizable), information on the label, BLAST results (species, accession number, score and % of identity with the best match), selling point (seller), inferred mislabeling cause (IUU as illegal, unreported and unregulated fishing) and inferred point in the commercial chain where the substitution took place (FM as fishmonger or retailer, SELL as selling point). Score < 500 marked as *. NA: not available. R, recognizable; U, unrecognizable.

Mislabeling was significantly more frequent in not recognizable products (10 out of 11 mislabeled products, Table 2), and in expensive species that are tuna and hake in this region (only two anchovies and none blue whiting). Mislabeling risk ratio RR of non recognizable versus recognizable products was 1.0369, 95% confidence [1.005–1.07], being significantly higher than 1 with z = 2.292 and *p* = 0.022. Similarly, mislabeling RR of expensive versus cheap species was 1.0371, 95% confidence [1.001–1.074], also significant (z = 2.022 with *p* = 0.043).

A deeper view at the mislabeled products (Table 2) suggests that the mislabeling found in this study could be compatible with IUU, at least in some cases. Mislabeled anchovies were about 12–13 cm long, and substitute species were small tuna fry or juveniles, one identified from DNA as yellowfin *Thunnus albacares* (the capture region stated on the label was NE Atlantic), and the other as *Thunnus alalunga* (captured from the Aegean Sea in the Mediterranean basin). The first one was sold whole, in fresh, eviscerated but with head and tail thus it was recognizable; the other was frozen headless, tailless and eviscerated. Under European (Regulation EU 850/98 of the European Council of 30 March, 1998) and Spanish laws (Royal Decree 560/1995 of 7 April, 1995), fish juveniles are protected and these tuna juveniles cannot be caught nor commercialized, thus the origin of these substitute fish was unequivocally IUU (Table 2). Small tuna fry and adult anchovies are indeed morphologically different but could be confounded by non-expert consumers, especially when sold headless. *Engraulis encrasicolus* distribution overlaps with spawning areas of *Thunnus albacares* in northeast African waters, and with those of *Thunnus alalunga* in the Mediterranean Sea, thus these substitutions are compatible with capturing tuna juveniles while fishing anchovy.

All the mislabeled hakes were sold frozen, in fillets or slices, labeled as *Merluccius capensis/M. paradoxus* that are South African hakes (Table 2). Three of them were substituted by *M. polli* that is an African species living from tropical and subtropical East Atlantic waters, and overlaps with *M. capensis* in the southern part of its distribution. South African *M. capensis/paradoxus* stocks are fished in a sustainable way certified by the Blue label of Marine Stewardship Council (https://www.msc.org/home/meet-the-wild-ones/from-sea-to-shelf-wild-south-african-hake, accessed January 2021), and they should not be caught together with other species. Thus the cause of this particular substitution is compatible with IUU (without discarding substitution in a later step). The other mislabeled hake was substituted by north Atlantic cod *Gadus morhua* which distribution does not overlap with south African hakes, thus substitution during fishing or landings was not possible in this case and must have occurred later in the commercial chain (Table 2).

Mislabeled tuna products were all labeled as “*bonito del Norte” Thunnus alalunga* (longfin or albacore tuna), which is the most frequently sold tuna species in the region. The three products that were substituted by northern Bluefin tuna *Thunnus thynnus* (Table 2) were all unrecognizable (slices), but the fish body (without the head) was visible and slices were cut fresh in front of the customer. They were sold in two different stores. The other two mislabeled samples did not report the capture region on the label. They were frozen slices, and were identified from DNA as yellowfin tuna *Thunnus albacares* and bigeye tuna *Thunnus obesus*, the last one with lower score and identity % with the best match in BLAST (Table 2). In the International Union for Conservation of Nature IUCN red list of threatened species^26^ *Thunnus thynnus* appears as globally endangered, *T. obesus* is vulnerable, and *T. alalunga* and *T. albacares* are near threatened. All the species overlap in the north—center east Atlantic and the EU has strict fishing quotas *T. thynnus* within a multiannual recovery plan (Regulation (EU) 2016/1627 of the European Parliament and of the Council of 14 September 2016).

### Fish consumption and its relation with mislabeling

In the social survey (N = 1608 participants aged between 14 and 63 years) the average frequency of fish consumption was 3.13 (SD 0.923), that is, surveyed participants declared to eat any fish with a frequency between monthly and weekly (1 being the highest and 5 the lowest frequency, 3 being weekly), that is, at least monthly. The raw dataset can be found in Rodriguez et al.^27^.

**Figure 1:**
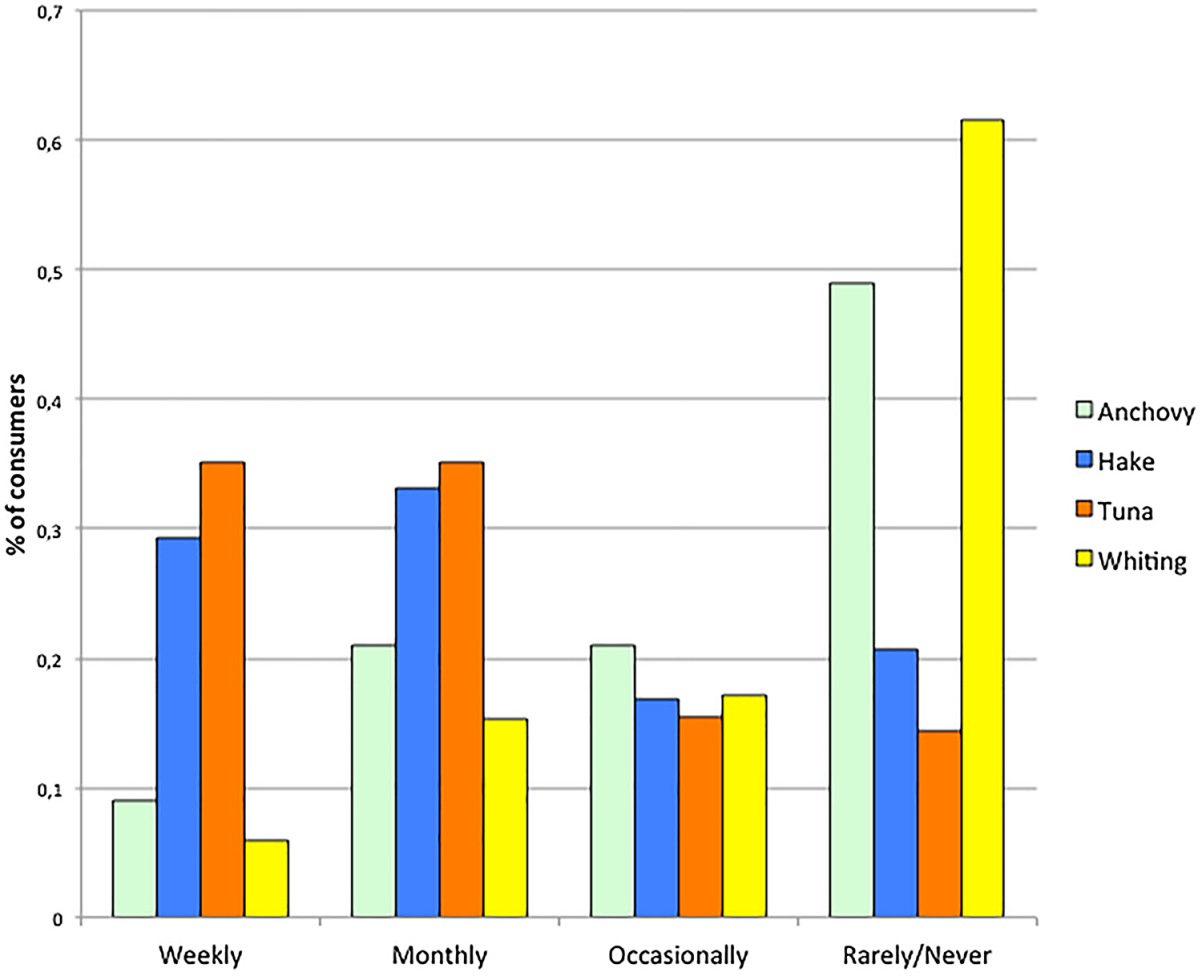
Consumption of the four species. Presented as the proportion of participants in the survey consuming a species weekly, monthly, occasionally or rarely/never.

A clear difference between species was found regarding consumption levels (Fig. 1). Tuna and hake were consumed weekly and monthly by a much larger proportion of participants than anchovy and whiting. Moreover, 49% and 61.6% of the participants declared not eating anchovy and blue whiting, respectively, being this proportion 20.7% for hake and 14.3% for tuna. The difference between species for the pattern of consumption frequency was highly significant ($\chi_{4,4}^{2}$ = 1154.8 with *p* <  < 0.001).

**Figure 2:**
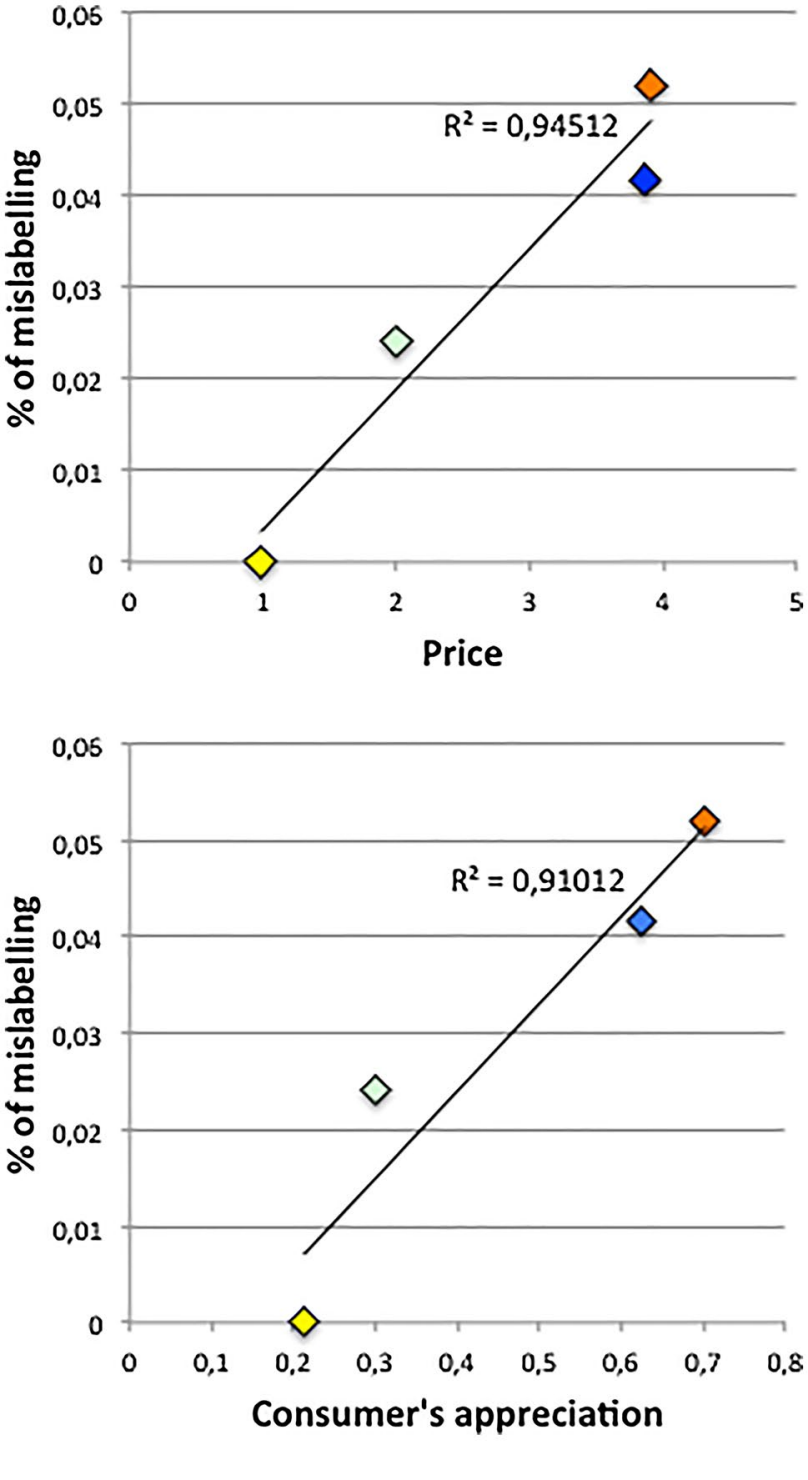
Plots of mislabeling on fish price (above), and on consumer’s appreciation (below). Results of consumer appreciation are presented as % of weekly and monthly consumers of a species. Linear trends with their r^2^ value for slope different from 0 are presented. Color codes: Anchovy = green; hake = blue; tuna = orange; whiting = yellow.

Consumer’s appreciation of a species was estimated from the proportion of participants consuming it at a frequency at least equal to the average frequency of fish consumption, this meaning that the species accounts for an important proportion of the fish consumed. Since the average fish consumption frequency in the region was 3.13 (= between monthly and weekly), we added the classes “Weekly” and “Monthly” (i.e. at least monthly) consumption of a species as a proxy of its appreciation. Mislabeling was significantly correlated with both price (Fig. 2 above) and the consumer’s appreciation proxy (Fig. 2 below): r^2^ = 0.945 and 0.91 respectively, both with *p* < 0.05.

**Figure 3:**
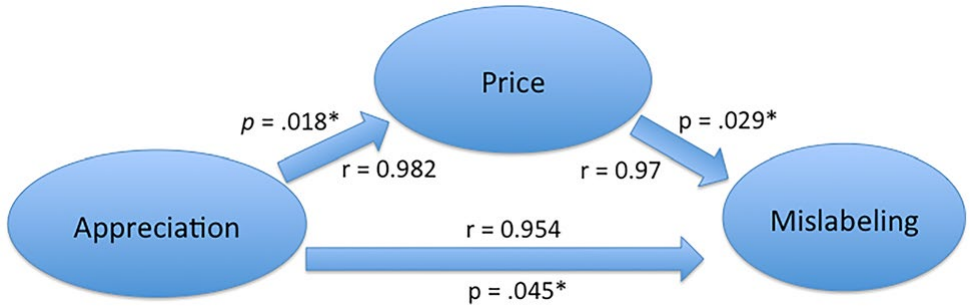
Model of price as a mediator between consumer’s appreciation price and mislabeling. Arrows represent relationships between variables. Pearson’s r coefficients and their p-values are given (* for *p* < 0.05).

The relationships between these variables are summarized in Fig. 3, with price as a mediator between consumer’s appreciation and fraudulent mislabeling. Zero-order unstandardized regression for predicting price from consumer’s appreciation (measured from consumption here) had B = 5.945 (SE = 0.806). Partial unstandardized regression coefficient for predicting mislabeling from fish price holding consumer’s appreciation constant was B = 0.015 with SE = 0.02 (Table 3). The distribution of products statistics Z_α_*Z_β_ was statistically significant being 5.376 > 2.18, *p* < 0.05. Mediation effect of price between consumer appreciation and mislabeling was thus significant in spite of limited number of species analyzed.

**Table 3:** Regression analysis of the variables mislabeling (dependent), price (expected mediator) and consumer’s appreciation (independent).

Variable	B	SE	t	*p*	R^2^
Constant (price)	− 0.038	0.407	− 0.092	.935	
Species appreciation	5.945	0.806	7.375	.018	0.965
Constant (mislabeling)	− 0.012	0.012	1.001	.499	
Price	0.015	0.020	0.729	.599	0.941
Species appreciation	0.003	0.122	0.026	.983	0.910

Unstandardized regression coefficients (B) and their standard error (SE), t statistics and its *p*-value, and R^2^ of the partial correlation. Zero-order regression predicting price from consumer’s appreciation above; below, multiple regression results of the three variables.

## Discussion

Combining a suite of molecular tools (three DNA markers and four different protocols) and a social survey amongst consumers, this work showed several evidences of fraud in appreciated species that are important for both food sciences and conservation. The mislabeling found in this study was likely fraudulent, since, according to hypothesis *a*, its risk was significantly higher in expensive than in cheap species, and in not recognizable than in recognizable products. Moreover, according to Hypothesis *b* it affected principally the species most appreciated by the consumer and amongst them those with higher prices, since price appeared to be a mediator between consumer’s appreciation (or, directly, consumption of a species) and mislabeling. This result points at the need of focusing on highly consumed highly priced species as priority mislabeling niche. Inadvertent mislabeling due to simple morphological confusion between species, frequently reported in previous studies in Spain^10^, does not seem the main cause of mislabeling here.

As in other studies, mislabeling occurred in tuna and hake. The level found was comparable to that published by other authors in Spain and elsewhere. Sotelo et al.^25^ found 6.7% mislabeling in fresh and frozen tuna in European markets of six countries between 2013 and 2014, similar to the 5.2% found here, although in Spain the level they found (20% in 20 samples) was clearly higher than in our study in which sampling was done six or five years later. Gordoa et al.^24^ also found higher levels of tuna mislabeling in Spanish supermarkets in samples of 2014–2015. It is possible that mislabeling is decreasing in Spain in the last years, following the current trend of improvement of market operations pointed out by Mariani et al.^6^ at European level. *Thunnus albacares* and *T. obesus* were the main substitutes in Sotelo et al.^25^ study, while *T. thynnus* was the most frequent substitute here. This reverse substitution (expensive *T. thynnus* for a cheaper species) has been rare, and when happening it was interpreted as indicative of illegal origin^24^. All together our results would indicate a change in tuna mislabeling trends in Spain.

Decrease of hake mislabeling seems to be happening in Europe as well, and the increase of the last years found in Blanco-Fernandez et al.^7^ review would be going down in Spain, since the proportion of mislabeled hake products found here (less than 5%) was low again. A difference with past mislabeling reports was the main substitute species found here, the Benguela hake *Merluccius polli*, rarely found as a substitute in other studies^7^. This would imply a change in hake substitutes and the unreported introduction of this species in European markets.

Anchovy mislabeling in Europe has been reported before^28^. It affects especially semipreserved products where it can as high as 16% being *Engraulis anchoita* the substitute species^29^. To our knowledge this is the first time that *Thunnus* is reported as a substitute of anchovy products. As in hake and tuna, it seems that the mislabeling trends are changing in this country towards different substitute species.

Although from our data and other studies^6^ fish mislabeling seems to be decreasing in Spain, the substitute species of this study are of concern for conservation because they point at the involvement of IUU, one of the causes of fraud identified by Fox et al.^20^, in current mislabeling in Spain. Finding small juveniles of near-threatened *Thunnus albacares* and *T. alalunga* sold as anchovy may reveal a serious problem in anchovy fisheries. If they catch juveniles of other species as by-catch, they are not sufficiently selective of the target species. Being true that the proportion was small, about 2.4% of the individuals analyzed, multiplying it by the tons of anchovy catch of Asturias fleet (1 566 059.4 kg in 2019 from official records; https://tematico.asturias.es/dgpesca/fich/992019_Z.pdf) it would represent about 37,585 kg of unreported tuna fry, more than 35 tons. On the other hand, the use of endangered *Thunnus thynnus* (and perhaps the vulnerable *Thunnus obesus* too) in substitution of less threatened tuna species is, definitively, a call of attention for the increase of control in European tuna markets, as demanded by other researchers^25^. Since the catch areas of label and substitute species overlap, these substitutions would suggest that Bluefin tuna caught over quota is being sold as another species.

Finally, *Merluccius polli* –main substitute of *M. capensis/paradoxus* in hake products here- is now the subject of sustainable management plan designs in its northern distribution^30^, while in the center and south the stocks are largely unknown^32^. Introducing the species as a substitute in European markets impedes traceability of its catch. It could be introduced at industrial level (for example to complete a batch of slices or fillets), or be another example of the use of unregulated fishing products. Selling *M. polli* from IUU would hamper the efforts of sustainable management of African hakes, both the currently MSC-certified South African *Merluccius capensis/paradoxus* and the future of Benguela hake stocks. introduction of the species as a substitute dustrial.

In conclusion, these results reaffirm a general trend of decreasing mislabeling rates, which is to be expected as efforts in food sciences for better traceability continue. However, while inadvertent substitutions decrease, special attention must be directed towards the detection of deliberate fraud. The found substitutes are an indication for IUUs as a source for fraud. This is a cause for concern and calls for a focus on products that are especially susceptible to these actions; i.e. highly consumed, expensive products. Monitoring of this type of products must be reinforced in order to prevent that fraudulent practices reach the consumer’s plate. Moreover, the whole SDG 12 is undermined if conscious consumers cannot be sure of eating really their choice of sustainably caught fish.

## Methods

### Ethics statement

This study adheres to good research practices based on the European code of conduct for research integrity^31^. Since part of the research required the application of a population survey, the Committee of Research Ethics of Asturias Principality approved this study with reference 166/19. All methods were performed in accordance with relevant guidelines and regulations. Prior to the application of the survey, all participants were provided with an information sheet detailing the objectives of the research and that no personal data, except age and place of application, would be collected. Informed consent was obtained from all the participants. Likewise, their willingness to participate in the research was guaranteed. They were invited to verify their answers, to have access to the study results, and were informed about their right to withdraw from the process at any time.

### Fish sampling

Fresh or frozen commercial products labeled as European anchovy (*Engraulis encrasicolus*), hake (genus *Merluccius*), tuna (genus *Thunnus*) and blue whiting (*Micromessistius poutassou*) were purchased from supermarkets, fishmongers and small shops in the region of Asturias (central south Bay of Biscay, Spain). Thus, they were products sold to consumers (final points in the commercial chain), all commercialized by European companies. Information about catch and prices of the four fish species in Asturias can be found in the General Directorate of Fisheries of Asturias Principality, https://tematico.asturias.es/dgpesca/din/estalonj.php (accessed in January 2021). Official statistics of fish prices in the region in 2019, when the samples were obtained, gave annual prices of 3.91, 3.89, 2 and 0.98 euro/kg of catch for tuna, hake, anchovy and blue whiting, respectively. Information about life cycles, distribution and spawning areas of these fish, and pictures, are available in FishBase website^32^ at www.fishbase.org.

Sampling was designed randomly from the list of stores selling fish in the region. It took place in 2019–2020. Fish products and labels were photographed and the information annotated in writing for double recording. Regulations (EU) No 1379/2013 of the European Parliament and of the Council of 11 December 2013 on the common organization of the markets in fishery and aquaculture products, and (EU) No 1169/2011 of the European Parliament and of the Council of 25 October 2011 on the provision of food information to consumers apply in Spain. From these regulations, seafood labels must contain at least the following information: common and scientific names of the species; geographic origin (country of production, or FAO area if from marine fishing); production method (extractive fisheries or aquaculture).

Products selected were big enough or contained the tail or head, to be sure that each sample corresponded to one individual. When the product was packed (e.g. fillets or slices) we sampled only one piece by pack. Products were classed as recognizable (i.e. the head is present and the whole body contains morphological characteristics that serve to identify the species) or unrecognizable (the species cannot be ascertained visually). Muscle fragments from each sample were cut, preserved in 100% ethanol and stored in the laboratory at 4 °C until analysis.

### Molecular analysis

DNA was extracted with Chelex-100 Resine (10%) and 4.5μL proteinase K (20 U/ml, by EURx) from frozen tissue. Different tools were employed for identification of the different fishes (Table 4). Cytochrome Oxidase Subunit I gene (COI) was employed for the first identification of blue whiting, anchovy and hake samples using primers described by Ward et al.^33^. Tuna was identified using the primer combination of L15998 with H-strand primer CSBDH, described by Alvarado-Bremer^34^ and used by Viñas and Tudela^35^ to distinguish between the eight *Thunnus* species from any kind of processed tissue, that amplify a fragment of the mitochondrial control region.

**Table 4:** Molecular tools employed in the analysis of fish fraud in this study, per species.

Region	Expected size	Primers	Reference	Annealing temperature	DNA in 20 μl	MgCl_2_	Species
COI	655 bp	COI-F1 5’- TCAACCAACCACAAAGACATTGGCAC-3’ COI-R1 5’- TAGACTTCTGGGTGGCCAAAGAATCA-3’	Ward et al. 2004	57 °C	2 μl	1.5 mM	Anchovy Blue whiting Hake
Control region	459 bp	L15998 5′-TAC CCC AAA CTC CCA AAG CTA-3′ CSBDH 5′-TGA ATT AGG AAC CAG ATG CCA G-3′	Alvarado-Bremer 1994	50 °C	4 μl	2.5 mM	Anchovy
						2 mM	Tuna
Control region	436 bp	MmerHK01 5’-GGGGGGGCCGACAGAGTTATA-3’ MmerHK02 5’-CCCGCTAGACTTGCTTACTAA-3’	Lundy et al. 2000	55 °C	2 μl	1.5 mM	Hake

In some cases, a second marker was necessary to distinguish between different closely related species belonging to the same genus. For anchovy, mitochondrial control region was amplified using the primers described by Alvarado-Bremer^34^. Mitochondrial control region was also amplified for the identification of hake samples, but using primers MmerHK01 and MmerHK02, described by Lundy et al.^36^.

PCR amplifications were carried out using 10 pmol of each primer, 1.5–2.5 mM MgCl_2_, 0.25 mM dNTPs, 1 × Buffer GoTaq Promega, 0.15 µl of GoTaq Polymerase (5u/µL) and 2-4µL of DNA in a final volume of 20µL, with little differences among primers (Table 4).

PCR amplifications were carried out in a thermo cycler ABI PCR 2700 (Applied Biosystems) with the following conditions: an initial denaturing step of 90 °C for 5 min, followed by 35 cycles consisting of 30 s of denaturing at 90 °C, 30 s of annealing at 55 °C for hake-control region amplification and 57 °C for COI, an elongation step for 45 s at 72 °C, and a final elongation step at 72 °C for 15 min after the cycles for hake-control region, and for 10 min for COI. And an initial denaturing step of 94 °C for 5 min, followed by 35 cycles consisting of 45 s of denaturing at 94 °C, 45 s of annealing at 50 °C, an elongation step for 1 min at 72 °C, and a final elongation step at 72 °C for 5 min for anchovy and tuna control region. Resulting amplicons were sent for Sanger sequencing to Macrogen Inc. (Spain).

Sequences were edited with BioEdit v.7.0.5.3. and Lasergene Seqman software by DNASTAR^37^. Sequences obtained from forward and reverse primers separately were combined whenever possible to obtain a more robust barcode. Edited sequences were compared to GenBank sequence database, using BLAST (Basic Tool Alignment Search Tool) (https://blast.ncbi.nlm.nih.gov/Blast.cgi) optimized for highly similar sequences (megablast), with word size = 28, match/mismatch scores of 1 and -2, linear gap costs, threshold 0.05. The best match hit was chosen as putative species of the sample from DNA.

### Survey to investigate consumers’ preferences

To know the habits and preferences regarding fish consumption, a survey was conducted in urban and rural geographical areas of Asturias. This Spanish province of 1,000,000 inhabitants has a rich tradition of fishing and seafood is important in its gastronomy. In a face-to-face format, the researchers went to secondary education centers, university schools/faculties and consumer centers for its application. Participants were asked to indicate their age, gender and place of residence. Two questions about fish consumption followed. The first question (five possible choices) was: “*How frequently do you consume fish normally? Please choose one of the following options: (1) More than 5 days a week; (2) Between 2 and 5 days a week; (3) One or two days a week; (4) Monthly; (5) Less than monthly”*. Then they were asked for the frequency of consumption of each of the four target species using the following question: *“How frequently do you eat the following species? Please choose the option that fits best your consumption habits”*, with four possible choices*: (1) Weekly; (2) Monthly; (3) Occasionally; (4) Rarely/Never”.*

### Statistics

To test hypothesis *a* about fraudulent mislabeling, comparison between different types of samples (e.g. recognizable versus not recognizable, cheap versus expensive species, etc.) regarding the proportion of mislabeled samples was done employing the risk ratio (RR), with z tests and their *p* H_o_ being RR = 1.

Differences in consumption between the four fish species based on the number of informers consuming a species weekly, monthly, occasionally or [rarely + never] (four classes, four species) were tested using Chi-square of contingency with nine degrees of freedom.

The variable *Consumer’s appreciation* of a species was estimated based on the average of total fish consumption. The proportion of participants that consume a fish species (anchovy, hake, tuna, blue whiting) at a frequency at least equal to the average frequency of any fish consumption (first question) was taken as a proxy of regional consumer’s appreciation of that species. Pairwise correlations between this variable, price and proportion of mislabeled products were calculated using Pearson’s r. The hypothesis of price as a mediator between consumer’s appreciation and mislabeling was tested from the distribution of products following MacKinnon et al.^38^ and Wuensch^39^, taking into account the small number of fish species where substitution was measured. Briefly, β = unstandardized regression coefficient for predicting the mediator (price) from the independent variable (consumer’s appreciation), and α = partial unstandardized regression coefficient for predicting mislabeling from price holding constant the consumer’s appreciation were divided by the standard errors to calculate Z_β_ and Z_α_ scores respectively. For a 0.05 non-directional test the critical value of the statistics Z_α_*Z_β_ is 2.18. Thus, higher values can be considered significant.

Significance threshold was *p* < 0.05. Statistics was done with free software PAST version 2.17c^40^.
